# Comparison between Intra-Articular Injection of Infrapatellar Fat Pad (IPFP) Cell Concentrates and IPFP-Mesenchymal Stem Cells (MSCs) for Cartilage Defect Repair of the Knee Joint in Rabbits

**DOI:** 10.1155/2021/9966966

**Published:** 2021-07-27

**Authors:** Yaguang Han, Haobo Li, Rong Zhou, Jun Wu, Ziye Liu, Huan Wang, Jiahua Shao, Yi Chen, Jun Zhu, Qiwei Fu, Qirong Qian, Yiqin Zhou

**Affiliations:** ^1^Department of Orthopedics, Shanghai Changzheng Hospital, Naval Medical University, Shanghai 200003, China; ^2^Department of Orthopedics, 72th Hospital of PLA, Huzhou, 313000 Zhejiang Province, China

## Abstract

Mesenchymal stem cells (MSCs) have emerged as a promising therapeutic method in regenerative medicine. Our previous research adopted a simple nonenzymatic strategy for the preparation of a new type of ready-to-use infrapatellar fat pad (IPFP) cell concentrates. The aim of this study was to compare the therapeutic efficacy of intra-articular (IA) injection of autologous IPFP cell concentrates and allogeneic IPFP-MSCs obtained from these concentrates in a rabbit articular cartilage defect model. IPFP-MSCs sprouting from the IPFP cell concentrates were characterized via flow cytometry as well as based on their potential for differentiation into adipocytes, osteoblasts, and chondrocytes. In the rabbit model, cartilage defects were created on the trochlear groove, followed by treatment with IPFP cell concentrates, IPFP-MSCs, or normal saline IA injection. Distal femur samples were evaluated at 6 and 12 weeks posttreatment via macroscopic observation and histological assessment based on the International Cartilage Repair Society (ICRS) macroscopic scoring system as well as the ICRS visual histological assessment scale. The macroscopic score and histological score were significantly higher in the IPFP-MSC group compared to the IPFP cell concentrate group at 12 weeks. Further, both treatment groups had higher scores compared to the normal saline group. In comparison to the latter, the groups treated with IPFP-MSCs and IPFP cell concentrates showed considerably better cartilage regeneration. Overall, IPFP-MSCs represent an effective therapeutic strategy for stimulating articular cartilage regeneration. Further, due to the simple, cost-effective, nonenzymatic, and safe preparation process, IPFP cell concentrates may represent an effective alternative to stem cell-based therapy in the clinic.

## 1. Introduction

Cartilage injury of the knee joint is a common condition of the locomotor system, which can lead to pain, restricted movement, and decreased joint function and may even gradually develop into knee osteoarthritis, thus representing a heavy burden to patients [1]. Since hyaline cartilage tissue on the articular surface is completely free of blood vessels, lymphatic vessels, and nerves, the articular cartilage has relatively poor self-repair and regeneration capabilities, complicating recovery following damage [2, 3]. The current treatments for articular cartilage regeneration mainly include drug and nondrug therapy as well as surgical treatment [4, 5]. Drug regimens include nonsteroidal anti-inflammatory drugs, chondroitin sulfate, and glucosamine, among other agents, while surgical interventions include microfracture (MF), allograft cartilage implantation, autologous chondrocyte implantation (ACI), scaffold-based tissue engineering techniques, and autologous cartilage chip (ACC) [6–9]. However, each method has certain limitations, such as limited therapeutic efficacy for drugs, small scale for microfracture method, and insufficient donor supply for osteochondral transplantation [10–14]. Hyaluronan has been widely used as viscosupplementation administered via IA injections, but debate over efficacy and safety continues. A 2015 systematic review concluded that no clinically relevant benefit was proven in terms of pain or function [15], and no convincing evidence of structural benefit is available. Intra-articular steroid injections are widely used to improve symptoms, but do not modify structure [16]. Therefore, none of these currently available treatment approaches can completely restore damaged articular cartilage [17].

In the past 20 years, mesenchymal stem cells (MSCs) have emerged as one of the most promising treatment methods for cartilage injury owing to their considerable self-renewal capacity, immunosuppressive ability, high plasticity, anti-inflammatory effects, and multidirectional differentiation potential [18–20]. Coculturing of MSCs with other cells could also provide a promising aspect in regenerative medicine, through providing the paracrine mediators, including growth factors and cytokines involved in the cross-talk between cells [21–26]. Since its first description in 2001, adipose-derived MSCs (ADSCs) have been considered as the most promising candidates for stem cell therapy due to their widespread availability and high regeneration potential [27–29]. The existence of infrapatellar fat pad- (IPFP-) MSCs in the knee joint was recently reported, and these are now being actively studied [30–34]. Although IPFP tissue and subcutaneous adipose tissue are both adipose tissue, IPFP-MSCs share embryonic homology with articular cartilage and are also located within the intra-articular environment [35]. A number of studies have confirmed that IPFP-MSCs possess excellent chondrogenic differentiation potential, having been successfully applied in osteoarthritis, articular cartilage injury, and other orthopedic diseases [35–38]. Further, they represent a favorable cell source for articular cartilage tissue engineering [39, 40]. In order to obtain these stem cells, a stromal vascular fraction (SVF) is generally prepared by enzymatic digestion of adipose tissue with collagenase or trypsin. However, possible concerns regarding enzymatic treatment, cell manipulation, and pathogen contamination have urged researchers to seek new solutions for obtaining adipose-derived products through a more accessible and feasible manner. To this end, ultrasounds and mechanical force have been employed to process adipose tissue using various devices [41].

In our previous research, we developed a new simple enzyme-free stem cell isolation technique [42]. Simply put, the autologous IPFP is mechanically processed and centrifuged to form a new type of ready-to-use IPFP cell concentrates with a short preparation time and without enzymatic digestion, additional cell expansion, or other complex manipulations. The IPFP cell concentrates provide a three-dimensional extracellular matrix environment as well as intrinsic IPFP-MSCs for the regeneration of articular cartilage. In our previous study, the short-term results were satisfactory and demonstrated that arthroscopic knee surgery with IPFP cell concentrates containing MSCs could reduce pain and effectively improve function in patients with knee cartilage defects, especially at 6 and 12 months after surgery [42]. However, to the best of our knowledge, there is no direct evidence comparing the efficacy of autologous IPFP cell concentrates and allogeneic IPFP-MSCs for the treatment of knee cartilage defects in an animal model. Therefore, it is important to evaluate whether the intra-articular (IA) injection of autologous IPFP cell concentrates achieves better therapeutic efficacy than allogeneic IPFP-MSCs obtained from these concentrates for articular cartilage regeneration.

## 2. Materials and Methods

### 2.1. Animals

All animal procedures and manipulations in this study were conducted following approval of the Committee on Ethics of Naval Medical University and in compliance with the Shanghai Changzheng Hospital Institutional Animal Care and Use Committee (IRB#: CZEC(2017)-13), following international guidelines for laboratory animal treatment. All skeletally mature female New Zealand white rabbits weighing 3 ± 0.5 kg (5–6 months) were obtained from the Laboratory Animal Centre of Naval Medical University (Shanghai, China). They were housed in individual steel cages, with unlimited activity within cages as well as free access to food and water. All rabbits were allowed to acclimatize for at least 10 days before surgical procedures and received standard postoperative antibiotic (5 mg/kg, cefazolin sodium pentahydrate, Gosun, Shenzhen, China) and analgesic regimens (4 mg/kg, carprofen, Rimadyl, Zoetis). Every effort was made to minimize animal suffering and the number of animals used. 36 rabbits were employed in the study, of which 18 were followed for 6 weeks and 18 were followed for 12 weeks. The rabbits used to create the cell lines are the same that then received the treatments out of consideration to reduce the number of rabbits used.

### 2.2. Preparation of IPFP Cell Concentrates

Our research team previously introduced a novel cell therapy-based strategy. During knee arthroscopic surgery, the surgeon used a standard electromechanical surgery blade, which is an arthroscopic device, to obtain part of the IPFP tissue. Briefly, about 200 mL of the mixture containing adipose tissue, synovial tissue, and normal saline was collected with the sterilized arthroscopic device of the collection system connected to a suction apparatus. The mixture was centrifuged at 300 g for 5 min after being filtered twice with a 30-mesh sterilized filter (pore size of 550 *μ*m) to remove the synovial tissue. 5 mL normal saline was used to resuspend the lipoaspirate for the preparation of IPFP cell concentrates after the liquid supernatant was discarded. However, considering that standard arthroscopic surgical instruments cannot be used on rabbit knee joints, we made some modifications to the IPFP cell concentrate preparation method. First, all rabbits were anesthetized by injecting tiletamine and zolazepam (5 mg/kg, Zoletil®50, Virbac, France) slowly into the ear vein. After adequate skin preparation and disinfection, an anterior-medium incision was made in the rabbit's right knee joint to expose the patella. Through the medial parapatellar approach, the patella was laterally dislocated to expose the distal femur's articular surface. The IPFP was obtained and dissected behind the patellar ligament with sterile ophthalmic scissors. Approximately 1 cm [3] of IPFP tissue was collected and finely minced using the scissors. The minced IPFP tissue was then transferred with normal saline to two 5 mL syringes connected by a luer-lock connector with an internal diameter of 2 mm. After 100 mechanical back-and-forth pushes, the chyliform fat was filtered to remove the connective tissue remnants and was centrifuged at 300 g for 5 min. After the liquid supernatant was discarded, 0.5 mL of normal saline was used to resuspend the lipoaspirate and was collected for further use. The cell surface antigen markers of IPFP cell concentrates were detected via flow cytometry.

### 2.3. Isolation and Culture of IPFP-MSCs

IPFP-MSCs were isolated from the previously extracted IPFP cell concentrates. Briefly, the obtained minced IPFP tissue was digested with 0.1% collagenase type I (Sigma-Aldrich) in PBS at 37°C under continuous shaking in a thermostatic oscillator at a speed of 120 rpm. After 45 min of digestion, low-glucose Dulbecco's Modified Eagle Medium (L-DMEM) (Hyclone) supplemented with 10% fetal bovine serum (FBS) (Gibco) and 1% antibiotics (Penicillin-Streptomycin) (Gibco) was used to terminate the enzymatic reaction. After tissue debris was removed by filtering through a 150 *μ*m cell mesh filter, the obtained cell filtrate was centrifuged for 5 min at 300 g. After discarding the supernatant, the harvested cell pellet was resuspended in L-DMEM/F12 (50% L-DMEM and 50% Ham's F-12) medium (Hyclone) supplemented with 10% FBS (Gibco) and 1% antibiotics (Penicillin-Streptomycin) (Gibco), seeded into a T-25 cell culture flask, and incubated at 37°C with 5% CO_2_ in a thermostatic incubator. The medium was changed every 2 days, and floating impurities were removed. The adherent cells were digested with 0.25% trypsin (Hyclone) upon reaching over 80% confluence and were subcultured at a ratio of 1 : 3. Cells of the third passage (P3) were used for subsequent experiments. The IPFP-MSCs were then subjected to flow cytometry (FC500, Beckman Coulter, USA) using a variety of antibodies against cell surface antigen markers (CD44, CD90, CD105, and CD45, BD Pharmingen, USA). Briefly, 100 *μ*L from isolated P3 cells was washed twice with phosphate-buffered saline (PBS) (Hyclone). The antibodies used for the identification of cell concentrates were CD44-PE-Cy, CD90-PE, PE-CD105, and CD45-PE. As a negative control, a cell suspension without antibodies was employed following the same procedure. The centrifuged cells were incubated with antibodies for 20 min at 4°C in the dark, resuspended in fluorescence-activated cell-sorting media, and analyzed immediately. In addition, MSCs were cultured for multilineage differentiation assays (osteogenesis, adipogenesis, and chondrogenesis), as previously described, in order to determine their differentiation potential. Briefly, IPFP-MSCs were seeded in a 6-well culture plate at a density of 2 × 10^5^ cells/well with osteogenic or adipogenic differentiation medium (Cyagen Biosciences, Guangzhou, China). After 21 days of culture, Alizarin red staining and Oil red O staining were performed to assess adipogenesis and osteogenesis potential, respectively. Micromass cell culture was performed for the chondrogenesis assay. A cell suspension droplet of 4 × 10^5^ cells was pipetted into a 15 mL centrifuge tube, and chondrogenic differentiation medium (Cyagen Biosciences) was then added. The medium was changed carefully once every 3 days. Alcian blue staining was performed to assess glycosaminoglycan formation after a 21-day chondrogenic induction, followed by 4% paraformaldehyde fixation and preparation of paraffin-embedded cartilage pellet sections.

### 2.4. Establishment of the Rabbit Articular Cartilage Defect Model

The initial steps for establishing an articular cartilage defect model are the same as the previously described surgical steps of obtaining IPFP. After exposing the trochlea of the distal femur, a cylindrical full-thickness cartilage defect (4 mm in diameter and 1.5 mm in depth) was induced on the trochlear groove using a sterile hand drill with the knee in full flexion. After carefully closing the surgical wound, a dose of intramuscular antibiotic (5 mg/kg, cefazolin sodium pentahydrate, Gosun, Shenzhen, China) was injected into the gluteus maximus once a day for three consecutive days to prevent infections. In addition, an intramuscular analgesic (4 mg/kg, carprofen, Rimadyl, Zoetis) was administered for postoperative pain control. The surgical site was disinfected with 0.1% povidone iodine every other day for two weeks. Wound healing was monitored, and no infection was observed. A total of 36 rabbits were randomly assigned to the following three treatment groups: the control group (treated with normal saline, *n* = 6), the IPFP cell concentrate group (*n* = 6), and the IPFP-MSC group (*n* = 6). Controls received an intra-articular injection of 0.5 mL normal saline. In the IPFP-MSC group, 1 × 10^7^ of IPFP-MSCs suspended in 0.5 mL normal saline were injected into the articular cavity. For the IPFP cell concentrate group, centrifuged lipoaspirate resuspended in 0.5 mL of normal saline was injected into the articular cavity.

### 2.5. Macroscopic and Histological Evaluation of Articular Cartilage Repair

After the rabbits were sacrificed with intravenous injection of Zoletil®50 at 6 and 12 weeks postsurgery, the distal portion of the femur was dissected and carefully photographed. The trochlear groove cartilage defect sites were then evaluated in a blinded manner by two independent observers based on the International Cartilage Repair Society (ICRS) scoring system (Supplementary Table 1). The distal femoral samples of rabbits were harvested at 6 weeks and 12 weeks, respectively, and were fixed in 4% paraformaldehyde solution for 48 h. The samples were then placed in 10% EDTA decalcification solution for 4-6 weeks, and the EDTA decalcification solution was changed every day. The acupuncture method was used to test the samples in order to determine whether decalcification has been completed. If the needle could be easily inserted into bone tissue, sample decalcification was considered completed. The decalcified samples were then dehydrated using a gradient concentration of ethanol, embedded in paraffin, and prepared into histological sections with a thickness of 4 *μ*m. The sections were stained with toluidine blue and Safranin O/Fast Green. ICRS visual histological assessment (Supplementary Table 2) was used to evaluate the histological repair of cartilage defects. Histological assessment was performed in a blinded manner by the same two independent observers.

### 2.6. Statistical Analysis

All statistical analyses were performed with the SPSS 22.0 software. All data were expressed as the mean ± standard deviation (SD). Sample size was set based on the results of other previous studies [43–45]. Comparisons were performed with the Student *t*-test or one-way ANOVA for experiments with more than two subgroups. Asterisks indicate significant differences (^∗^*P* < 0.05; ^∗∗^*P* < 0.01) compared with the corresponding control. Kappa statistics were applied to intraobserver variations with values between 1.00 and 0.81 indicating perfect accord, between 0.80 and 0.61 indicating substantial accord, between 0.60 and 0.41 indicating moderate accord, between 0.40 and 0.21 indicating fair accord, between 0.20 and 0.00 indicating slight accord, and below 0.00 indicating poor accord.

## 3. Results

### 3.1. Multilineage Differentiation Potential and Surface Marker Expression of Cell Concentrates and Pure MSCs Derived from IPFP

**Figure 1:**
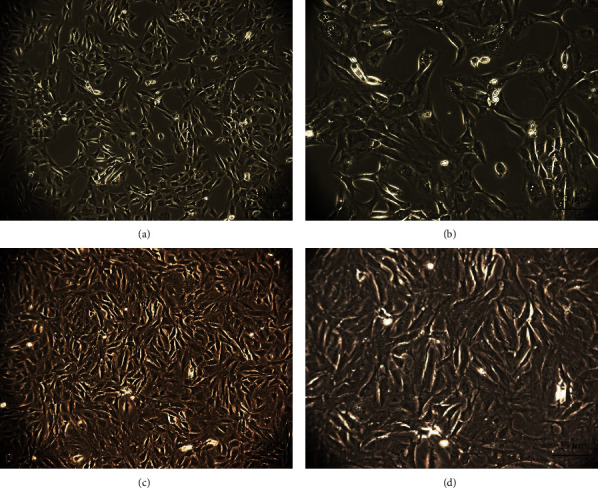
The microscopical appearance of the third passage generation of IPFP-MSCs from the (a, b) IPFP-MSC group and the (c, d) cell concentrate group exhibited typical spindle-shaped morphology and cells were anchorage dependent.

Primary IPFP-MSCs were isolated using the aforementioned method and observed via light microscopy every day thereafter. Cells from the IPFP-MSC group and the cell concentrate group were cultured for 21 days, and representative cells were photographed under a light microscope. Cells from both groups exhibited typical spindle-shaped morphology and were anchorage dependent (Figure 1).

**Figure 2:**
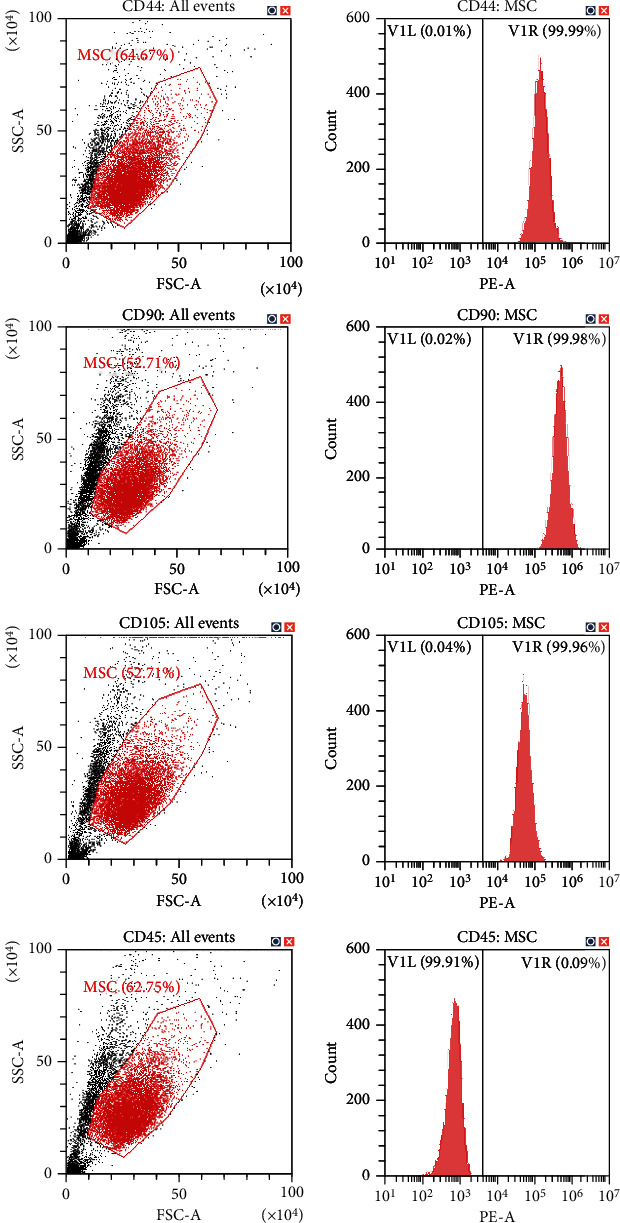
The surface markers of IPFP-MSCs isolated from three rabbits characterized by flow cytometry in passage 3: no expression of CD45 and strong expression of CD44, CD90, and CD105.

**Figure 3:**
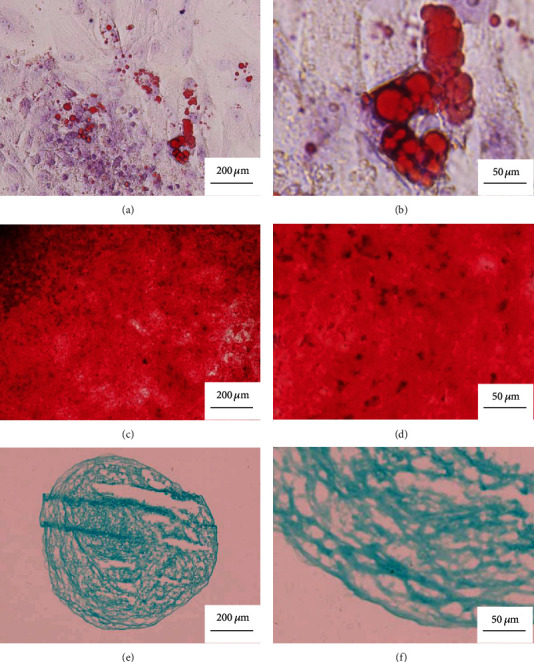
The three-way differentiation and staining results of IPFP-MSCs isolated from three rabbits. (a, b) After staining with Oil red O for adipogenic differentiation, red lipid droplets fused into pieces can be seen. (c, d) After staining with Alizarin red for osteogenic differentiation, there were red calcium nodules fused into masses. (e, f) After staining with Alcian blue for chondrogenic differentiation, a large amount of blue-stained acid mucopolysaccharide appears among the cells.

According to the results of flow cytometry, an average of (1.03 ± 0.87) × 10^7^ cells/mL of cell concentrate were harvested from each rabbit of the IPFP cell concentrate group, while (4.93 ± 2.74) × 10^5^ cells/mL of cell concentrate exhibited MSC marker expression (CD45-, CD44+, CD90+, and CD105+), with an average percentage of 5.07 ± 2.69%. As for the IPFP-MSC group, flow cytometry indicated that 99% of cells expressed CD44, CD90, and CD105, while <1% of cells expressed CD45 (Figure 2). With regard to the multilineage differentiation potential test, positive Alcian blue, Alizarin red, and Oil Red O staining confirmed that MSCs successfully differentiated into chondrocytes, osteocytes, and adipocytes, respectively (Figure 3).

### 3.2. Gross Evaluation of Cartilage Repair

**Figure 4:**
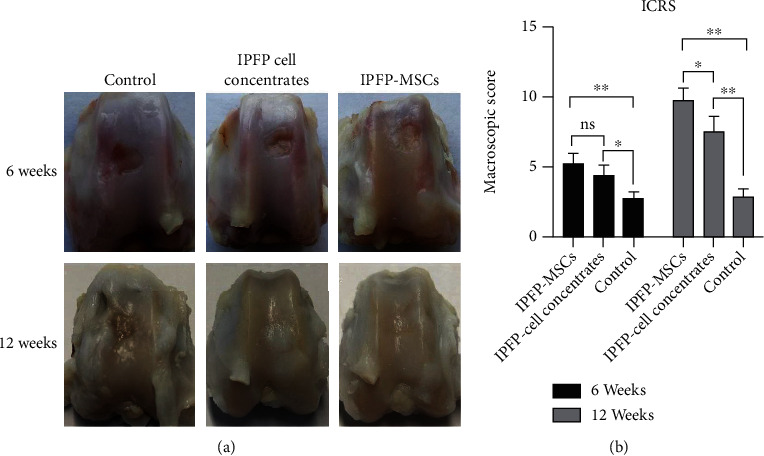
(a) The gross appearance of cartilage repair in vivo at 6 and 12 weeks after surgery. (b) The ICRS macroscopic evaluation scores of articular cartilage repair. (All data were expressed as the mean ± standard deviation (SD). Comparisons were performed with the Student *t*-test or one-way ANOVA for experiments with more than two subgroups. Asterisks indicate significant differences (^∗^*P* < 0.05; ^∗∗^*P* < 0.01) compared with the corresponding control).

As shown in Figure 4(a), 6 weeks after intervention, the IPFP cell concentrate group exhibited hyperplastic tissue on injured cartilage, with obvious surface indentation and a clear boundary around the injured section. In contrast, more hyperplastic tissue on the injured cartilage was observed in the IPFP-MSC group, with slight surface indentation and a visible boundary around the injured section. No hyperplastic tissue was observed in the control group. 12 weeks after intervention, the injury sites in the IPFP cell concentrate group exhibited enhanced tissue regeneration, yet still retained a rough surface and recognizable boundary. In the IPFP-MSC group, greater regenerated tissue was observed, exhibiting a smooth surface integrated within the surrounding area and having color and texture similar to those of normal cartilage. Obvious surface indentation and a boundary with fibrotic tissue were observed around the border of injury sites in the control group.

The ICRS scoring results corresponded to macroscopic appearance. As shown in Figure 4(b), the ICRS scores of the IPFP-MSC group (5.25 ± 0.75) were not significantly higher (*P* > 0.05) than those in the IPFP cell concentrate group (4.42 ± 0.73) after 6 weeks, while both experimental groups had significantly higher scores (*P* < 0.05) than the control group (2.75 ± 0.48). At 12 weeks, the ICRS scores in the IPFP-MSC group (9.83 ± 0.80) were significantly higher (*P* < 0.05) than those in the IPFP cell concentrate group (7.58 ± 1.02). Both experimental groups were significantly higher (*P* < 0.05) than the control group (2.92 ± 0.53).

### 3.3. Histological Assessment of Cartilage Repair

**Figure 5:**
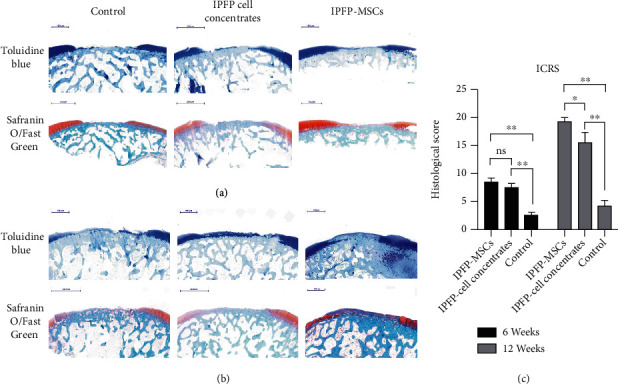
(a, b) Toluidine blue and Safranin O/Fast Green staining of different groups at 6 and 12 weeks. (c) The ICRS visual histological assessment scores of articular cartilage repair. (All data were expressed as the mean ± SD. Comparisons were performed with the Student *t*-test or one-way ANOVA for experiments with more than two subgroups. Asterisks indicate significant differences (^∗^*P* < 0.05; ^∗∗^*P* < 0.01) compared with the corresponding control).

Cartilage repair was assessed by observing tissue sections at 6 weeks after operation (Figure 5(a)). In IPFP-MSC group specimens, the bottom of the original cartilage defect area was covered with a new cartilage layer, which was relatively continuous but uneven. The new cartilage was mainly fibrocartilage, and no typical hyaline cartilage was observed. In the IPFP cell concentrate group, the bottom of the defect area was covered with a small amount of new tissue, which was discontinuous. There was no obvious chondrocyte production and no obvious filling in the concave area. In the blank control group, no new tissue was formed in the defect area, and subchondral bone destruction was observed. There was a clear boundary between the defect area and the surrounding cartilage tissue in all groups.

At 12 weeks after the operation, the regenerative effect was greater than that observed at 6 weeks for both experimental treatments (Figure 5(b)). Defect areas in the IPFP-MSC group were completely filled with new tissue, and the surface was smooth. The new tissue was connected to surrounding cartilage tissue and was dominated by fibrocartilage. In the IPFP cell concentrate group, the bottom of the defect area was covered with a new layer of fibrous cartilage, concave, and its surface was uneven. Surfaces in the blank control group were also uneven, with fibrous connective tissue proliferation and irregularity observed.

The cartilage repair histological score was assigned to 6 specimens per group at 6 weeks and 12 weeks after operation. At 6 weeks, the IPFP-MSC group had a score of 8.58 ± 0.61, the IPFP cell concentrate group averaged 7.50 ± 0.65, and the control group had a score of 2.58 ± 0.53. There were no significant differences in score (*P* > 0.05) between the IPFP-MSC group and the IPFP cell concentrate group, while both had significantly higher scores (*P* < 0.05) than the control group, as shown in Figure 5(c). At 12 weeks after operation, the IPFP-MSC group had an average score of 19.25 ± 0.85, the IPFP cell concentrate group had a score of 15.67 ± 1.62, and the control group average was 4.25 ± 0.99. Significant differences (*P* < 0.05) were observed between all groups, as shown in Figure 5(c).

## 4. Discussion

In this study, we successfully extracted cell concentrates containing IPFP-MSCs by nonenzymatic method and pure IPFP-MSCs by enzymatic method from IPFP. After identifying the multilineage differentiation potential and surface marker expression of cell concentrates and pure MSCs, further in vivo study was launched to evaluate their efficacy on the repair of cartilage defects. The results were encouraging and demonstrated that both IPFP-MSCs and cell concentrates derived from IPFP could facilitate the regeneration of articular cartilage defects. Although IPFP cell concentrates were not as efficient as IPFP-MSCs in repairing articular cartilage damage, they exhibited the simple, efficient, cost-effective, safe, and nonenzymatic preparation process, thus providing a novel cell-based therapy approach for the clinic.

Cartilage defects have a very limited intrinsic healing capacity. With the lack of blood and lymph vessels as well as nerves, large cartilage defect regeneration occurs only through the production of fibrous tissue or fibrocartilage [46]. Quality articular hyaline cartilage repair remains an unmet need for treatment of cartilage injury to prevent progression into osteoarthritis (OA) [47, 48]. Stimulating regeneration of articular cartilage by penetration of the subchondral bone with techniques such as microfracture has been a common procedure for treating cartilage injury in clinical practice. However, long-term durability was inadequate, as the native hyaline cartilage was repaired with fibrocartilage consisting of primarily type I collagen, which is less adaptable than its hyaline counterpart and fails to provide a long-term therapeutic effect, and in some cases, it even results in deterioration of the joint function and leads to progression into OA [49–52]. It is thought that microfracture alone does not recruit a sufficient amount of reparative cells and growth factors to promote adequate native cartilage repair [53, 54]. In this study, we used a novel nonenzymatic strategy to obtain autologous ready-to-use cell concentrates derived from knee IPFP adipose tissue containing MSCs. We then compared cell concentrates with a pure stem cell-based traditional enzymatic approach with regard to the multilineage differentiation potential of cellular components and the therapeutic efficacy for articular cartilage restoration.

MSCs, as cells with multipotent differentiation potential, have become a major focus within the field of cartilage regeneration. Recent studies have proven that MSCs derived from bone marrow and adipose tissue can be acquired in large amounts for clinical use [19, 55–59]. Compared with bone marrow-derived MSCs, those from adipose tissue exhibit greater proliferative capacity, and their acquisition causes less damage to the donor zone [60, 61]. In recent years, MSCs derived from IPFP adipose tissue, which are particularly easy to acquire in large amounts with minor damage and exhibit great proliferation and differentiation capacities, have emerged as an ideal cellular resource for stem cell-based knee articular cartilage repair. As substantial clinical trial evidence has confirmed the safety of IPFP-MSCs in knee cartilage defects, alleviating pain, and improving knee function [62, 63], extraction in a safe, fast, and effective manner has become a major factor for promoting their application in the clinic.

Thus far, two major IPFP-MSC-based approaches for the treatment of knee articular cartilage damage have been explored, namely, therapy with MSC-containing cell concentrates and pure stem cell therapy. In general, enzymes are applied during the MSC extraction process, contributing to higher costs, complicating manipulation, and potentially affecting safety as well as efficacy [56, 64, 65]. Considering the economic and safety-related implications of traditional enzymatic digestion-based extraction methods, researchers have focused on nonenzymatic procedures using centrifugation, filtration, and microfragmentation. However, given the diverse protocols of such nonenzymatic procedures and the lack of further evaluation in animal experiments and clinical trials, conflicting data have been provided regarding the efficacy of these methods. Further, the percentages of karyocytes and progenitor cells in concentrates extracted via most nonenzymatic methods are obviously lower compared to those obtained through enzymatic methods. Researchers believe that this is due to more MSCs localizing around vascular structures within adipose tissue without enzyme digestion. Thus, harvesting more karyocytes and progenitor cells by mechanical methods, such as mechanical vibration, washing-up, and centrifugation with different parameters, has become a major focus. Raposio et al. isolated cell concentrates through a nonenzymatic method under a laminar air flow bench without using serum or animal-derived reagents [66]. The results indicated that only 5% of the cells were progenitors, and the concentrate was insufficient for demonstrating that the obtained cells were indeed MSCs. According to Aronowitz et al., while cell concentrates isolated through enzymatic methods usually contain 15% progenitor cells, the isolation process takes from 30 min to 2 h, in contrast to no more than 15 minutes required for nonenzymatic methods [67]. In the current study, we isolated cell concentrates from rabbit IPFP via a nonenzymatic method, which included mechanical vibration and adipose emulsification, thus simulating the extraction method of cell concentrates during knee arthroscopy in humans.

Based on standards proposed by the International Society for Cellular Therapy (ISCT) in 2006, MSCs should meet the following criteria: (1) cell adhesion growth in vitro; (2) positive expression of CD44, CD73, CD90, and CD105 (positive detection rate of flow cytometry > 95%) and negative expression of CD11b, CD14, CD19, CD34, CD45, CD79a, and HLA-DR (the positive detection rate of flow cytometry < 2%); and (3) ability to differentiate into osteoblasts, adipocytes, and cartilage cells in vitro under specific culture conditions [68]. In the current work, according to the results of flow cytometry, an average of (1.03 ± 0.87) × 10^7^ cells/mL of cell concentrate was harvested from each rabbit of the IPFP cell concentrate group, while (4.93 ± 2.74) × 10^5^ cells/mL of cell concentrate exhibited MSC marker expression (CD45-, CD44+, CD90+, and CD105+), with an average percentage of 5.07 ± 2.69%. As for the IPFP-MSC group, flow cytometry indicated that 99% of cells expressed CD44, CD90, and CD105, while <1% of cells expressed CD45 (Figure 2). Moreover, cells from concentrates extracted without enzymatic digestion as well as pure cells isolated enzymatically were both successfully induced to differentiate into osteoblasts, adipocytes, and chondrocytes in vitro.

In our study, even though MSCs in cell concentrates isolated via the nonenzymatic method had lower purity compared pure stem cells, they exhibited significant efficacy in cartilage repair compared to control treatment. This may be due to the local microenvironment established by cell concentrates, which in turn affect the differentiation of MSCs. On the other hand, they could also modify it releasing paracrine mediators in order to restore tissue homeostasis [30, 59, 69]. According to previous studies, MSC proliferation and differentiation are influenced in a paracrine manner by cytokines and growth factors released by the grafted cells, triggering host-associated signaling pathways, increasing angiogenesis, and suppressing apoptosis [70, 71].

With regard to clinical application, adipose tissue can be sourced from the IPFP of the synovial joint environment during knee arthroscopic surgery. In our previous prospective, randomized control study with a 12-month follow-up period, knee arthroscopic therapy with IPFP cell concentrates containing MSCs was proven to be safe, reducing pain and improving joint function in patients with knee cartilage defects [42]. Further, as cell concentrates were derived from the IPFP, which is usually partially removed or “abandoned” during regular arthroscopy surgery, it may represent a kind of waste recycling, significantly reducing the cost of treatment and thus increasing patient compliance.

Nevertheless, the present study has some other limitations. The first is that the number of cells in concentrates to be injected was limited and not equal among the rabbits. Second, the sample size was small, and the follow-up period was short. Thus, a study with more animals and a relatively longer observation period needs to be carried out in the future. Last, the underlying molecular mechanism of cartilage repair requires further investigation.

Although the nonenzymatic method for isolating MSC-containing cell concentrates from the IPFP is at the initial stage of testing, tissue-engineering techniques with MSC-containing concentrates hold promise for damaged joint cartilage repair in clinic.

## 5. Conclusions

In conclusion, the present study demonstrated that IPFP-MSCs and cell concentrates derived from IPFP could facilitate the regeneration of articular cartilage defects. The cell concentrates were minimally manipulated lipoaspirate that retains a certain amount of extracellular matrix and MSCs after mechanical processing. Concentrates were not as efficient as IPFP-MSCs in repairing articular cartilage damage, but still exhibited good repair efficacy. Considering the simple, efficient, cost-effective, safe, and nonenzymatic preparation process of IPFP cell concentrates, they represent a promising therapeutic approach for articular cartilage regeneration, providing a novel cell-based therapy approach for the clinic.

## Data Availability

The original data is available from the corresponding author Yiqin Zhou (drzhouyiqin@163.com).

## Supplementary Materials

Supplementary Materials Supplementary Table 1: the ICRS macroscopic evaluation of cartilage repair. Supplementary Table 2: the ICRS visual histological assessment scale. Supplementary Table 3: the ARRIVE Essential 10: Compliance Questionnaire.
