# Supplementary material for: Comparison between Intra-Articular Injection of Infrapatellar Fat Pad (IPFP) Cell Concentrates and IPFP-Mesenchymal Stem Cells (MSCs) for Cartilage Defect Repair of the Knee Joint in Rabbits
Source: Stem Cells Int. 2021 Jul 27;2021:9966966. doi: 10.1155/2021/9966966 (PMC8337123; doi:10.1155/2021/9966966)
Supplement: Supplementary Materials — Supplementary Table 1: the ICRS macroscopic evaluation of cartilage repair. Supplementary Table 2: the ICRS visual histological assessment scale. Supplementary Table 3: the ARRIVE Essential 10: Compliance Questionnaire. [file 9966966.f1.docx]

**Supplementary Table 1. The ICRS macroscopic evaluation of cartilage repair**

| **Categories** | **Scores** |
| --- | --- |
| **I. Degree of defect repair** |  |
| In level with surrounding cartilage | 4 |
| 75% repair of defect depth | 3 |
| 50% repair of defect depth | 2 |
| 25% repair of defect depth | 1 |
| 0% repair of defect depth | 0 |
| **II. Integration to border zone** |  |
| Complete integration with surrounding cartilage | 4 |
| Demarcating border < 1 mm | 3 |
| 3/4 of graft integrated, 1/4 with a notable border > 1 mm width | 2 |
| 1/2 of graft integrated with surrounding cartilage, 1/2 with a notable border > 1 mm | 1 |
| From no contact to 1/4 of graft integrated with surrounding cartilage | 0 |
| **III. Macroscopic appearance** |  |
| Intact smooth surface | 4 |
| Fibrillated surface | 3 |
| Small, scattered fissures or cracks | 2 |
| Several, small or few but large fissures | 1 |
| Total degeneration of grafted area | 0 |
| **Overall repair assessment** |  |
| Grade I: normal | 12 |
| Grade II: nearly normal | 11–8 |
| Grade III: abnormal | 7–4 |
| Grade IV: severely abnormal | 3–1 |

The International Cartilage Repair Society (ICRS) macroscopic scoring assessment scale for cartilage repair (scale of 0 to 12, with 0 being severely abnormal cartilage and 12 indicating normal cartilage)

**Supplementary Table 2. The ICRS visual histological assessment scale**

| **Features** |  | **Scores** |
| --- | --- | --- |
| **Surface**  Smooth/continuous  Discontinuities/irregularities  **Matrix**  Hyaline  Mixture: hyaline/fibrocartilage  Fibrocartilage  Fibrous tissue  **Cell distribution**  Columnar  Mixed/columnar-clusters  Clusters  Individual cells/disorganized  **Cell population viability**  Predominantly viable  Partially viable  <10% viable  **Subchondral bone**  Normal  Increased remodeling  Bone necrosis/granulation tissue  Detached/fracture/callus at base  **Cartilage mineralization (calcified cartilage)**  Normal  Abnormal/inappropriate location  **Toluidine blue stain**  Normal  Slight reduction  Moderate reduction  Severe reduction  No staining  **Percent toluidine blue in defect**  75–100%  50–75%  25–50%  0–25%  No toluidine blue staining |  | 3  0  3  2  1  0  3  2  1  0  2  1  0  3  2  1  0  2  0  4  3  2  1  0  4  3  2  1  0  Max 24 |

The International Cartilage Repair Society (ICRS) visual histological score for cartilage repair (the highest score is applied to the ideal repair result (i.e., truly regenerated tissue), and the lowest score is applied to the poorest repair result)

**Supplementary Table 3. The ARRIVE Essential 10: Compliance Questionnaire**


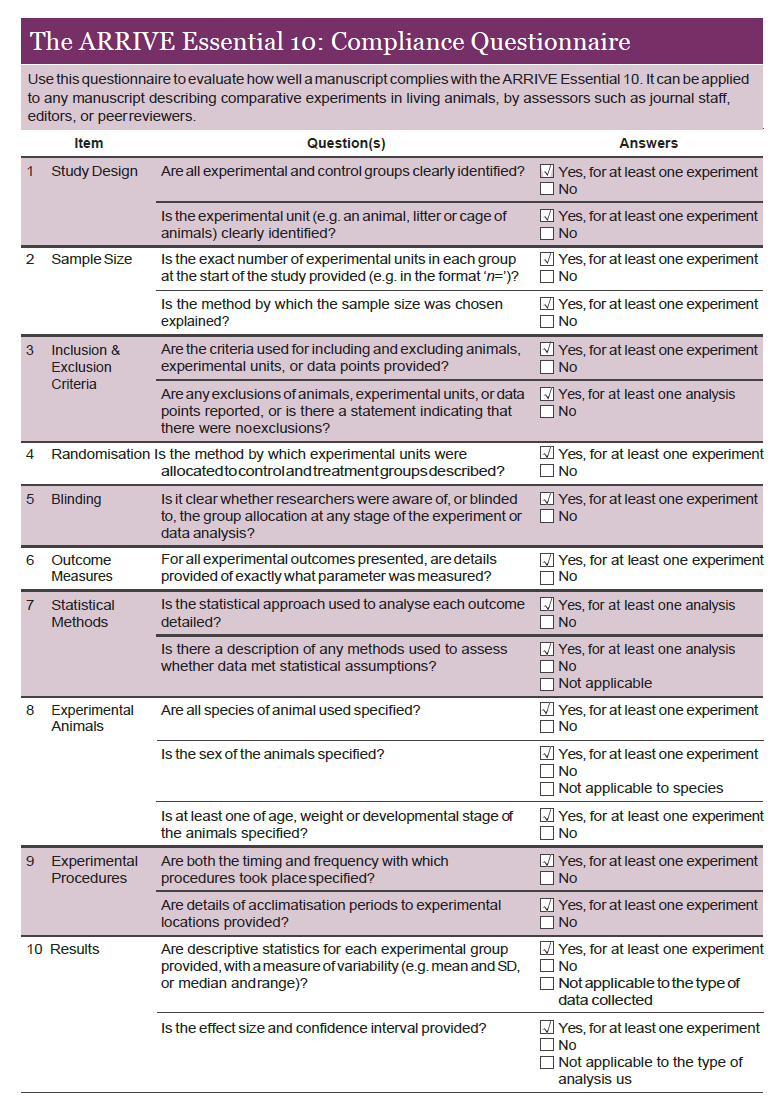


The ARRIVE guidelines (Animal Research: Reporting of *In Vivo* Experiments) are a checklist of recommendations to improve the reporting of research involving animals – maximising the quality and reliability of published research, and enabling others to better scrutinise, evaluate and reproduce it.
